# The Mental Suffering of Cocaine-Addicted Patients: A Retrospective Analysis of Personality Disorders’ Prevalence and Their Association with Psychopathological Symptoms

**DOI:** 10.3390/ejihpe14100183

**Published:** 2024-10-14

**Authors:** Francesca Giordano, Sara Guidotti, Francesco Cassio Scategni, Domenico Cuzzola, Carlo Pruneti

**Affiliations:** 1 Service for Pathological Addictions, Department of Pathological Addictions, National Health Service of Lecce, 73100 Lecce, Italy; francesca_giord_23@libero.it (F.G.); cassio.scategni@libero.it (F.C.S.); domcuzz@yahoo.it (D.C.); 2 Clinical Psychology, Clinical Psychophysiology, and Clinical Neuropsychology Laboratories, Department of Medicine and Surgery, University of Parma, 43126 Parma, Italy; carlo.pruneti@unipr.it

**Keywords:** cocaine, addiction, personality disorder, psychopathological symptoms, substance abuse

## Abstract

(1) Background: The observational retrospective study aimed to investigate the prevalence of personality disorders (PDs) and their association with psychopathological symptoms in a group of patients with cocaine addiction. (2) Methods: Ninety-five medical records of the Pathological Addictions Service of the National Health Service of Lecce (Italy) were analyzed. PDs were diagnosed using the Structured Clinical Interview for DSM-IV Axis II Personality Disorders (SCID-II) and psychopathological symptoms were investigated through the Symptom Checklist 90-Revised (SCL-90-R). (3) Results: Ninety-two out of ninety-five patients met the criteria for at least one PD (96.8%), almost 35% had two Cluster B PDs, and over 25% had three Cluster B PDs. Nine out of ninety-five people met the criteria for all Cluster B PDs. Among the Cluster B PDs, it emerged that the most frequent diagnosis was that of narcissistic–borderline–antisocial (over 20% of the total sample). The analysis highlighted that mental suffering is prevalent in those with multiple comorbid Cluster B PDs. Specifically, depression and psychoticism exceed the clinical cut-off (T score > 63) in all patient groups. At the same time, anxiety and obsessions–compulsions are complained of only by those with more than two PDs as well as the level of general distress. Furthermore, anxiety, hostility, and paranoid ideation are significantly higher in the group of patients with more than three PDs. (4) Conclusions: Further studies should better investigate the relationship between the two aspects and describe the causal effects of PDs on psychopathological symptoms or, on the contrary, the effects of the substance on mental health and the worsening of personality alterations.

## 1. Introduction

As reported by the latest estimates from the European Drug Report [1], approximately 83 million people have used illicit substances during their lifetime, equal to 28.9% of the European adult population. The average age for starting abuse/addiction is around 34 years globally, although the age of onset is rapidly moving forward from 23 years to early adolescence. Considering the population of young adults (15–34 years), there are 15.8 million people using cannabinoids, 2.2 million for cocaine, 2.0 million for MDMA, and 1.4 million for amphetamines.

Nevertheless, there are indications that cocaine has become more readily accessible in the European market. Over the past two years of social restrictions due to lockdowns, the use of crack cocaine significantly increased, particularly among younger generations [2,3]. National values fluctuated between 0.2% to 4.6% of the young Italian population regarding cocaine use, being so prevalent to be second only to the consumption of cannabinoids [1].

The DSM-5-TR [4] describes substance-related disorders, dividing them into two groups: substance use disorders (SUDs) (addiction and abuse) and substance-induced disorders (SIDs) (intoxication, withdrawal, and other substance/medication-induced mental disorders). The essential feature of a SUD is a cluster of cognitive, behavioral, and physiological symptoms (i.e., impaired control, social impairment, risky use, and pharmacological criteria) and constant use despite significant related problems.

Furthermore, the different substances produce specific induced mental disorders during intoxication and abstinence [5]. To illustrate, sedating substances (i.e., sedatives, hypnotics, anxiolytics, and alcohol) can cause significant depressive disorders during intoxication, while anxiety conditions are more frequent during abstinence. On the contrary, stimulant substances (i.e., amphetamines and cocaine) are more likely associated with substance-induced psychotic and anxiety disorders during intoxication and major depressive episodes during abstinence [4,6,7]. Both conditions are associated with significant, although transitory, sleep disorders and sexual dysfunctions, while neurocognitive symptoms, especially affecting executive functions, are long-lasting [8].

Because SUDs and other mental disorders have a high co-occurrence rate, research proved that the chances of suffering from a psychiatric disorder among substance users are around 3–4 times higher than in the general population [9]. Additionally, more than a third of individuals affected by a mental disorder also struggle with psychoactive substance abuse or addiction [10]. On the other hand, about 70% of individuals abusing cocaine have a personality disorder (PD), with antisocial (ASPD) and borderline (BPD) being the most prevalent [11]. Research attested that substance-use-related psychiatric disorders and PDs are associated with worse psychosocial and medical status during treatment, too [12,13]. Nevertheless, BPD seems to be linked to attempted suicide, needle sharing, and more serious psychopathology [14], such as depressive disorders and alcoholism [13]. Studies comparing drug addicts with and without BPD found that those with BPD had a greater tendency to abuse substances throughout their lifetime and scored high on impulsivity measures [15,16,17,18]. Patients with BPD are more prone to experiencing cravings and relapses triggered by negative emotional states, tension, social rejection, and negative physical states [17]. Prospective studies found that impulsivity is a stable factor over time and highly predictive of borderline psychopathology over seven years of follow-up [19].

In light of these assumptions, it was deemed useful to update data concerning the prevalence of PD among patients with cocaine-induced SUD and the association with psychopathological symptoms. Our retrospective observational study analyzed patients’ medical records in the care of an Italian Pathological Addictions Service, containing psychological questionnaires administered for clinical purposes. The data analysis was guided by the hypothesis that a high prevalence of PDs would be found in association with clinically relevant psychopathological symptoms.

## 2. Materials and Methods

### 2.1. Participants

In this retrospective observational study, the researchers analyzed data from ninety-five medical records of patients who accessed the Pathological Addictions Service of the National Health Service of Lecce (Italy) between 2018 and 2023. Criteria for inclusion in the study were age > 18 years old; completion of informed consent; no history of psychiatric and neurological syndromes (e.g., previous head trauma, epilepsy, etc.) and physical diseases (i.e., sensory disturbances of sight and hearing) that might have limited the administration of the questionnaires; medical diagnosis of SUD for stimulants (cocaine) at first visit; and absence of concomitant heroin use.

### 2.2. Procedure

The administration of the psychological assessment took place at the time of access to the service, after confirmation of the medical diagnosis of SUD of the stimulant type (cocaine), before entering any treatment, both psychological and integrated.

The Ethical Committee of the Hospital of Lecce approved the study (protocol number: 61/2023). All procedures were conducted following the 1964 Declaration of Helsinki and the 2005 Universal Declaration on Bioethics and Human Rights regarding research involving human participants.

### 2.3. Measures

The psychopathological symptoms were investigated through the Symptom Checklist 90-Revised (SCL-90-R) [20]. The SCL-90-R is a self-report instrument measuring the presence and extent of internalizing and externalizing psychopathological symptoms experienced by the participant in the week preceding the compilation. It includes 90 items referring to the following different clinical scales: Somatization (SOM; 12 items) (α = 0.89), Obsessive and Compulsive (O-C; 10 items) (α = 0.93), Interpersonal sensitivity (I-S; 9 items) (α = 0.85), Depression (DEP; 13 items) (α = 0.95), Anxiety (ANX; 10 items) (α = 0.92), Hostility (HOS; 6 items) (α = 0.88), Phobic anxiety (PHOB; 7 items) (α = 0.87), Paranoid ideation (PAR; 6 items) (α = 0.86), and Psychoticism (PSY; 10 items) (α = 0.79). The Global Severity Index (GSI) (α = 0.87) is the best indicator of an individual’s disorder’s current level or depth. It combines information concerning the number of symptoms reported with the intensity of perceived distress. It is obtained by adding the scores of all 90 items and dividing by 90. The items are rated on a five-point Likert scale of distress in the previous week: “Not at all” (0), “A little bit” (1), “Moderately” (2), to “Quite a lot” (3), and “Extremely often” (4). The raw score is obtained from the total score of each scale divided by the number of its items. The authors suggest that a T score for the GSI above 63 points or a T score of any two symptom dimensions above 63 points generally indicates a significant clinical psychological problem [21]. The symptom dimensions have Cronbach’s alpha indices between 0.67 (PHOB) and 0.87 (DEP), which can be considered acceptable to excellent.

The PDs were assessed through the Structured Clinical Interview for DSM-IV Axis II Personality Disorders (SCID-II) [22,23], an interview divided into three parts. The initial phase of the interview process requires the interviewee to complete a self-administered questionnaire consisting of 120 questions regarding their personality traits. The completion of the questionnaire typically takes around 20 min and is a prerequisite for the interview. Failure to complete this questionnaire may disqualify an individual as a suitable candidate for the SCID-II, as the interview hinges on their ability to acknowledge their personality traits. The interview involves a leaflet detailing criteria related to personality traits, which can be rated as absent (score 1), subthreshold (score 2), or present (score 3). Each criterion corresponds to one question from the initial questionnaire. If an interviewee responds positively to a question in the self-administered questionnaire, the corresponding criterion is further discussed with the interviewer, following the instructions in the second part of the leaflet. Moreover, the questionnaire answers can also be discussed if a certain personality trend becomes apparent during the interview. The third part of the interview involves recording demographic data and the total scores on separate papers that come with the SCID-II.

### 2.4. Statistical Analysis

All of the statistical analyses were performed using SPSS (Version 28.0.1.0; IBM Co., Armonk, NY, USA). First, descriptive statistics were performed with the calculation of the mean (M) and standard deviation (SD). Tests for skewness and kurtosis and the Kolmogorov–Smirnov test were used to determine the normality of distribution. In a multicollinearity test, no extreme coefficient values ≥0.8 were found among the independent variables, indicating a low risk of multicollinearity. All independent variables had variance inflation factors ≤10 and tolerance ≥0.1, indicating the absence of multicollinearity.

Then, to test our hypothesis, the only group of patients diagnosed with Cluster B PDs was considered. A comparison of socio-demographic (i.e., age, sex, marital status, and current occupation) and psychopathological symptoms (i.e., SCL-90-R T scores) between groups divided by the number of PD diagnoses (1 PD group, 2 PDs group, and 3–4 PDs group) was made through the conduction of chi-squared test (after Yates correction for small samples, effect size was defined through Cramer’s V and considered weak if it was ≤0.2, moderate if it fell between 0.2 and 0.6, and moderate if it was >0.6) and two-way ANOVAs with the consequent calculation of partial eta-squared (η^2^ = 0.01 relates to a small effect, η^2^ = 0.06 indicates a medium effect, and η^2^ = 0.14 or higher indicates a large effect), respectively. Gender and age were included as covariates in the two-way ANOVAs. Post hoc analyses on clinical scales of SCL-90-R were obtained through the Bonferroni correction.

Lastly, Pearson’s correlation analysis was conducted to examine the association between psychopathological symptoms (i.e., somatization, obsessive and compulsive, depression, etc.) and personality traits (i.e., histrionic, narcissistic, borderline, and antisocial) as well as gender and age.

## 3. Results

### 3.1. Descriptive Statistics and Correlations between the Variables of Interest

The sample was composed of 11 (11.6%) females and 84 (88.4%) males aged between 18 and 55 years old (M_age_ = 34.33, SD_age_ = 8.82). Most of the participants were married (56.68%) (33.68% were unmarried, 12.63% were separated or divorced) and employed (59.11%) (31.58% were unemployed, 6.32% were students). In addition, all subjects voluntarily accessed the Pathological Addictions Service and received a medical diagnosis of SUD by cocaine. Most of the patients took cocaine intranasally (61 people, 66.3%), while 31 of them took it by smoking (i.e., crack) (32.6%), and 1 (1.1%) by injection. All patients were visiting the service for the first time and had never been treated by other mental health services.

As concerns the PD diagnoses, ninety-two (96.84%) people met the criteria for at least one of them. More specifically, twenty-five (26.32%) of them met the criteria for only one PD. Three (3.16%) people satisfied the criteria for antisocial, nine people (9.47%) for borderline, two people (2.11%) for histrionic, seven people (7.37%) for narcissistic, three people (3.16%) for obsessive-compulsive, and one person (1.05%) for paranoid. Then, thirty-three people (34.74%) had two PDs. Nine people (9.47%) had borderline–antisocial, two people (2.11%) had histrionic–antisocial, three people (3.16%) had histrionic–borderline, three people (3.16%) had histrionic–narcissistic, four people (4.21%) had narcissistic–antisocial, and twelve people (12.63%) had narcissistic–borderline. Additionally, a group of twenty-five people (26.32%) had three comorbid PDs. One person (1.05%) had histrionic–borderline–antisocial, two persons (2.11%) had histrionic–narcissistic–antisocial, two persons (2.11%) had histrionic–narcissistic–borderline, and twenty people (21.05%) had narcissistic–borderline–antisocial. Lastly, nine people (9.47%) met the criteria of all four Cluster B PD diagnoses.

Only patients with at least one Cluster B PD diagnosis were included to test our hypotheses. The sample (*n* = 88) was divided into three groups based on whether they had one, two, or three or more Cluster B PDs. Descriptive statistics are reported in Table 1. There were no significant differences between groups when looking at the socio-demographic variables. Although it did not reach statistical significance, a slight imbalance between groups was observed for marital status, with a weak/moderate effect size.

Looking at the psychopathological symptoms, Table 2 presents the mean and standard deviation of each clinical scale of the SCL-90-R for each sample group. Moreover, scores indicative of the presence of psychopathology (T score > 63) were found in the “2 PDs group” and “3–4 PDs group” for anxiety and only in the “3–4 PDs group” for hostility and paranoid ideation. The “3–4 PDs group” reported pathological values in all the clinical scales, except for interpersonal sensitivity, although it approached clinical relevance. Pathological values are present in all three groups for both depression and psychoticism. The ANOVA shows significant differences between the groups for anxiety, hostility, and paranoid ideation. A medium effect was attested for anxiety and paranoid ideation, while hostility reached a large effect size. The only significant covariate was gender (coded as 0 = female and 1 = male) for hostility (gender: F(1) = 12.41, *p* < 0.001), which indicated higher levels among females.

Post hoc analyses demonstrated that having three or more PDs was associated with increased anxiety, hostility, and paranoia compared to those meeting the criteria for only one PD. Specifically, levels of anxiety, hostility, and paranoia (controlled for gender and age) were significantly higher in the “3–4 PDs group” compared to the “1 PD group”. Furthermore, the difference in hostility between the “2 PDs group” and the “3–4 PDs group” approached statistical significance (Table 3).

### 3.2. Correlations between Variables

Looking at the correlations between the variables of interest, it emerged that all of the psychopathological symptoms were significantly associated with each other. Furthermore, anxiety, hostility, and paranoid ideation were confirmed to be positively correlated with the number of PD diagnoses. Looking at the personality traits, the borderline trait correlated with all of the psychopathological symptoms. Hostility was associated with the narcissistic trait and paranoid ideation with both the narcissistic and the antisocial traits.

Lastly, mood alterations, such as depression and hostility, were negatively correlated with the male gender as well as the antisocial trait. The borderline trait was positively associated with the female gender (Table 4).

## 4. Discussion

The present study aimed to investigate the prevalence of PD, analyzing clinical records of a group of patients who were in the care of an Italian pathological addictions service during the period from 2018 to 2023. Consistently with the literature, it emerged that almost all of the subjects were males [16] and met the criteria for at least one PD (only three subjects reported the absence of comorbid PD) [24]. Among the remaining patients, almost 35% had two Cluster B PDs, and over 25% had three Cluster B PDs. Indeed, nine out of ninety-five people met the criteria for all four Cluster B PDs. Nevertheless, the high prevalence of Cluster B PDs attested was even higher than those reported by other authors. As already described in a 1995 review, the percentage of cocaine-using patients with comorbid PD is between 30% and 100%, with differences attributable to the diagnostic tools used. Particularly, clinical observation ranges from 31 to 90%, Millon Clinical Multiaxial Inventory from 97 to 100%, and SCID-II from 31 to 74%, with higher sensitivity to be attributed to the more recent clinical instruments used to assess personality. More recently, other authors found a prevalence of PDs between 20 and 25% using the SCID-II [24,25]. Specifically looking within the categories (1 PD, 2 PDs, and 3–4 PDs groups), it emerged that the most frequent diagnosis is that of narcissistic–borderline–antisocial (over 20% of the sample), followed by narcissistic–borderline (more than 10%), borderline–antisocial (almost 10%), and histrionic–narcissistic–borderline–antisocial (almost 10%), confirming the presence of the borderline trait as the one most frequently found in comorbidity with SUD by cocaine [25,26,27,28,29,30].

As already found by Verheul [31], ASPD and BPD were the most widespread PD diagnoses, although our total percentage exceeded the one reported previously (almost 75% of our patients met the criteria for antisocial and borderline while the literature described 20% in total). The prevalence of PDs among cocaine addicts has likely increased following the COVID-19 pandemic, which exacerbated several other mental disorders [32,33].

As regards the psychopathological symptoms investigated through the SCL-90-R, the descriptive analysis highlighted scores worthy of clinical interest. In cocaine use disorder, psychopathological symptoms may manifest during acute intoxication, chronic use, and withdrawal, in which depression, anhedonia, paranoia, and impaired judgment, with potentially dangerous impulsive behavior, may greatly intensify [6,34]. A depressive mood alteration was observed in all three groups (T scores > 63) of our sample. According to research, depressive symptoms may be related to neurobiological damage induced by the chronic consumption of stimulants, which favor a reduction in dopamine neurotransmission [35]. The danger of mood alteration is associated with the loss of the possibility of experiencing pleasure, with even greater exposure to the risk of suicide [36] due to the crash state [37]. However, they are partially reversible as psychobiological mechanisms explained by epigenetics mediating their manifestation [5]. Similarly, symptoms associated with psychoticism exceeded the clinical cut-off of the SCL-90-R for all of the patients groups of our sample, confirming the so-called substance-induced psychosis (SIP) [7,34,38,39] described several times among people abusing substances with higher psychotomimetic properties (i.e., cannabis, cocaine, and amphetamines) [40,41]. Even though paranoia is also a consequence of excessive and continuous use of stimulants [42], pathological levels were reached only by the group of patients with three or more PDs, in which such ideation was significantly higher than in the other patients’ groups. A similar trend was also observed for anxiety and hostility levels with moderate to large effects. In this regard, women experience greater levels of psychopathological comorbidity due to biological differences between the sexes as well as greater social stigma connected to SUDs [10,24], as it emerged that gender was the only significant covariate on hostility.

Consistently with the psychopathological aspects described above, chronic substance abuse is often accompanied by a progressive loss of emotional–relational bonds, social isolation, and a worsening reduction in work functionality [43], as evidenced by our sample characterized by a high percentage of unemployed (approximately one in three patients regardless of the number of PD diagnoses). These conditions may favor the further maintenance of depressive experiences and self-devaluation, which increase the probability of implementing poorly self-preserving behaviors (i.e., suicidal and parasuicidal behavior, sharing needles with a higher risk of contracting HIV infection, liver-related disease, drug-related arrests, and incarceration) [44].

Another salient aspect to underline concerns emotional dysregulation, which plays a fundamental role in the predisposition to SUD as well as in the maintenance of the disorder and the recovery of global functioning even after remission [16,18,45]. To illustrate, neuroimaging studies [15,46] corroborated that a marked alteration of recognition and management of emotions favored the dual diagnosis (cocaine addiction and Cluster B PD).

However, it should be taken into consideration that the correlation analysis evidenced that the borderline trait measured by the SCID-II was the only one to be significantly associated with all of the psychopathological symptoms, confirming previous studies regarding higher levels of psychopathology among cocaine-addicted BPD patients [13,17]. Consistent with these assumptions, previous scientific studies demonstrated that traits connected to BPD are, in turn, linked with substance abuse. For instance, diagnostic manuals (i.e., DSM-5-TR) consider potentially dangerous behaviors, including the abuse of psychoactive substances, as a possible criterion to make the diagnosis. In a study investigating the relationship between BPD, ASPD, and the prediction of harm among substance users [14], it was found that the levels of impairment in the dual group (BPD + ASPD) were identical to BPD, confirming the fundamental role of the borderline trait in provoking mental suffering. It seems that the aspects of impulsivity and disinhibition are the crucial point that connects BPD and, more generally, Cluster B PDs’ alterations with substance addictions, in which the drug dependence would be associated with the so-called “disinhibitory personality trait” [47]. To give an example, several researchers proved that there are brain alterations (i.e., frontal-executive deficits) supporting these traits [15,16].

Nonetheless, to our knowledge, there are no studies that have analyzed the differences between groups of subjects with Cluster B PDs based on the number of comorbid PDs. Looking at the subdivision created, it was observed that having “only” one PD is not enough for the experiencing of clinically significant distress in terms of psychopathological symptoms assessed through the SCL-90-R (the GSI T score was found to be lower than 63). On the other hand, patients diagnosed with two or more PDs complained of mental distress over the clinical cut-off (GSI T score > 63). On top of this, looking at the significant differences calculated between the groups, it emerged that the group of patients with three or more PDs complained of even higher levels of anxiety, hostility, and paranoia compared with the “1 PD group”. These data could be in line with the self-medication theory [48,49], according to which taking a psychoactive substance favors the management of some emotional state perceived as intolerable. Then, one could hypothesize that patients suffering from three or four comorbid Cluster B PDs may have reported higher levels of psychopathology as a result of a highly disturbed personality. It is also possible to assume that subjects with three or more PDs abuse to a greater extent and have, as a result, more cocaine-induced psychopathological symptoms.

In any case, the fact that anxious activation reached the clinical cut-off of the SCL-90-R only among patients with two or more PDs is crucial. As emotional activation is an indicator of suffering and, consequently, of motivation to change [50], its absence may not be a good indicator of adherence to treatment and its prognosis. Being aware of the dangers of psychoactive drugs must make the early identification of users a priority, with consequent treatment aimed at both the SUD and the underlying PD. By way of illustration, cocaine was assigned second place (preceded only by heroin) for its level of harmfulness [51]. Although the maximum age for cocaine addiction is between 23 and 25 years old, once use begins, it develops much more rapidly and “explosively” in comparison with marijuana or alcohol. By way of illustration, up to 5–6% of cocaine users will develop addiction within the first year of use [52].

In summary, our study highlighted how the prevalence of Cluster B PDs among cocaine-addicted patients is high and, above all, how the most frequent clinical condition is characterized by the copresence of narcissistic, borderline, and antisocial traits. For the first time, it was underlined that patients who satisfy the criteria for only one PD do not report significant psychopathological symptoms and that only those who have two or more comorbid PDs manifest psychological distress. This aspect interferes with good practices for taking care of drug-addicted patients because it does not facilitate their approach and motivation to change [53].

Notwithstanding, our findings must be interpreted in light of the present limitations. Since the research is a retrospective observational study, it is not possible to clearly define the directionality of the data. For instance, the psychopathological symptoms complained of by patients with two or more Cluster B PDs may not only be the consequence of personality alterations and the substance taken and abused. Conversely, it may be possible that PDs are exacerbated by SUD and that continued cocaine use may be the cause of a large portion of personal and interpersonal dysfunction. As previously mentioned, when referring to the disinhibitory personality trait, it is important to keep in mind that the predisposition to SUD by cocaine and the development and maintenance of the spectrum of Cluster B PD traits share the same neural substrates and that the emotional–behavioral manifestations probably fall along a continuum that is difficult to categorize. Considering that neither the chronological course of the diagnoses nor the duration and severity of cocaine abuse were measured, these aspects remain among the main limitations of the research conducted.

Future studies will need to take into account the chronological course of the diagnosed conditions (e.g., age of onset). By definition, PDs should emerge during adolescence or early adulthood, but the consequences (psychological, behavioral, and social) of SUD may mimic the diagnostic criteria of cluster B PDs. Since the effects of the substance may constitute a major confounding factor, future research will need to measure the severity and duration of cocaine abuse. Interesting results could also emerge by investigating the form of administration with personality traits. On top of this, further studies could isolate the different PDs as well as syndromic disorders (i.e., anxiety, depression, and paranoia), to create subgroups of similar size and make appropriate comparisons. A better-balanced sample for socio-demographic variables (i.e., gender, marital status) could overcome the limitations connected to the type of sample analyzed in the present study (i.e., convenience sample). Lastly, future studies could replicate the procedures presented in this work by including additional measures for the assessment of psychopathological symptoms (i.e., Structured Clinical Interview for DSM-5 Disorders-Clinician Version) that can overcome the limit of self-administration of questionnaires such as the SCL-90-R, sensitive to the subjective perception of one’s level of suffering. To illustrate, instruments that contain valence scales able to intercept the tendency to amplify or diminish one’s mental suffering (i.e., Minnesota Multiphasic Personality Inventory) could also be useful in this regard.

Notwithstanding, the line of studies of the individual characteristics that predispose and favor stimulant use disorders (such as cocaine and crack) must be enriched despite the arduous nature of the task. Since the presence of PD comorbid with SUD is associated with a worsening of the integrity of some brain functions (such as that of the orbitofrontal cortex, necessary for emotional processing and elaboration) [18], it is essential to intercept the traits that could interfere with the benefit of the proposed treatment [12]. The dramatic increase in demand for treatment has made it impossible to wait for the results of controlled research to guide pathological addiction services, and the identification of early intervention programs represents one of the most critical problems facing the substance abuse treatment field today.

## 5. Conclusions

Our retrospective research analyzed the prevalence of PDs in a group of patients who were treated by an Italian drug addiction service between 2018 and 2023. Our data attested to a higher percentage of PD among cocaine addicts compared to what was previously reported in the literature. As cocaine SUD and Cluster B PDs are intrinsically connected, the study of the various components affecting the dual diagnosis is not easy. To conclude, dealing with pathological addictions involves the management of this complexity, as toxicological, psychopathological, interpersonal–social, and educational–cultural aspects are included. Further research is necessary to address the intertwining of variables and analyze how trait characteristics influence SUDs and psychopathological symptoms. Pathological addiction services require updated lines of intervention. Guidelines that describe the patient’s level of well-being or, on the contrary, discomfort are required to delve deeper into thoughts and behaviors that need to be modified and emotional regulation that has to be supported.

## Figures and Tables

**Table 1:** Comparisons of socio-demographic features between groups.

Variable	1 PD Group (*n* = 20)	2 PDs Group (*n* = 33)	3–4 PDs Group (*n* = 34)	ANOVA or Chi-Squared Test	*p*	Cramer’s V or η^2^
Age, *M* (*SD*)	35.4 (9.3)	34.3 (8.5)	34.1 (9.3)	F(2) = 0.14	0.87	0.003
Sex, *N* (%)				χ^2^ (2, *N* = 88) = 1.62	0.45	0.14
Male	17 (81%)	30 (90.9%)	31 (91.2%)			
Female	4 (19%)	3 (9.1%)	3 (8.8%)			
Marital status, *N* (%)				χ^2^ (4, *N* = 88) = 9.12	0.06	0.23
Single/Unmarried	17 (81%)	17 (51.5%)	14 (41.2%)			
Married/Cohabitant	2 (9.5%)	11 (33.3%)	15 (44.1%)			
Divorced/Widowed	2 (9.5%)	5 (15.2%)	5 (14.7%)			
Current Occupation, *N* (%)				χ^2^ (4, *N* = 88) = 0.53	0.97	0.06
Student	2 (9.5%)	2 (6.1%)	2 (5.9%)			
Employed	12 (57.1%)	21 (63.6%)	20 (58.8%)			
Unemployed	7 (33.3%)	10 (30.3%)	12 (35.3%)			

**Table 2:** Comparisons of psychopathological symptoms between groups.

Variable	1 PD Group (*n* = 20)	2 PDs Group (*n* = 33)	3–4 PDs Group (*n* = 34)	ANOVA	*p*	η^2^
Somatization	55.0 (13.6)	60.2 (17.5)	64.0 (25.2)	F(2) = 1.26	0.29	0.03
Obsession–Compulsion	61.2 (16.5)	66.3 (18.2)	69.5 (19.8)	F(2) = 1.26	0.29	0.03
Interpersonal Sensitivity	58.9 (19.3)	59.3 (15.4)	62.7 (17.4)	F(2) = 0.43	0.65	0.01
Depression	63.5 (20)	66.9 (18.6)	70.2 (23.1)	F(2) = 0.67	0.52	0.02
Anxiety	59.2 (16.1)	65.6 (20.2)	74.5 (25.1)	F(2) = 3.42	0.04	0.08
Hostility	51.7 (10.2)	60.1 (17.1)	71.4 (24.9)	F(2) = 6.94	0.002	0.14
Phobic Anxiety	53.6 (8.7)	55.0 (14.5)	69.5 (21.4)	F(2) = 1.37	0.26	0.03
Paranoid Ideation	59.0 (16.9)	62.1 (15.5)	74.9 (21.4)	F(2) = 6.21	0.003	0.13
Psychoticism	70.2 (24.7)	69.9 (24.7)	80.2 (28.4)	F(2) = 1.56	0.22	0.04
Global Severity Index	62.5 (18.3)	67.1 (18.5)	74.7 (25.3)	F(2) = 2.27	0.11	0.05

Note: Data are presented as mean (standard deviation).

**Table 3:** Post hoc comparison analysis of ANOVA’s effect.

	Post Hoc				Limit	
		Mean Difference	SE	*p*	Lower	Upper
Somatization	1 PD > 2 PDs	−5.2	5.7	1.00	−19.2	8.8
	1 PD > 3–4 PDs	−9.0	5.7	0.35	−22.9	4.9
	2 PDs > 3–4 PDs	−3.8	4.9	1.00	−15.8	8.2
Obsession–Compulsion	1 PD > 2 PDs	−5.0	5.2	1.00	−17.8	7.8
	1 PD > 3–4 PDs	−8.3	5.2	0.35	−21.0	4.5
	2 PDs > 3–4 PDs	−3.3	4.5	1.00	−14.3	7.8
Interpersonal Sensitivity	1 PD > 2 PDs	−0.4	4.9	1.00	−12.2	11.5
	1 PD > 3–4 PDs	−2.7	4.8	1.00	−15.5	8.1
	2 PDs > 3–4 PDs	−3.4	4.2	1.00	−13.6	6.9
Depression	1 PD > 2 PDs	−3.4	5.9	1.00	−17.8	11.0
	1 PD > 3–4 PDs	−6.7	5.9	1.00	−11.0	17.8
	2 PDs > 3–4 PDs	−3.3	5.1	1.0	−15.7	9.1
Anxiety	1 PD > 2 PDs	−6.5	6.1	0.88	−21.3	8.4
	1 PD > 3–4 PDs	−15.3	6.1	0.04	−30.1	−0.5
	2 PDs > 3–4 PDs	−8.8	5.3	0.29	−21.7	4.0
Hostility	1 PD > 2 PDs	−8.4	5.5	0.40	−21.8	5.1
	1 PD > 3–4 PDs	−19.8	5.5	0.002	−33.1	−6.4
	2 PDs > 3–4 PDs	−11.4	4.8	0.06	−23.0	0.2
Phobic Anxiety	1 PD > 2 PDs	−1.4	4.7	1.00	−12.9	10.2
	1 PD > 3–4 PDs	−6.8	4.7	0.45	−18.3	4.7
	2 PDs > 3–4 PDs	−5.4	4.1	0.56	−15.4	4.5
Paranoid Ideation	1 PD > 2 PDs	−3.2	5.2	1.00	−15.9	9.5
	1 PD > 3–4 PDs	−16.0	5.2	0.008	−28.6	−3.3
	2 PDs > 3–4 PDs	−12.8	4.5	0.02	−23.8	−1.9
Psychoticism	1 PD > 2 PDs	0.35	7.4	1.00	−17.8	18.5
	1 PD > 3–4 PDs	−10.0	7.4	0.54	−18.5	17.8
	2 PDs > 3–4 PDs	−10.3	6.4	0.33	−26.0	5.3
Global Severity Index	1 PD > 2 PDs	−4.6	6.1	1.00	−19.5	10.2
	1 PD > 3–4 PDs	−12.2	6.0	0.14	−27.0	2.5
	2 PDs > 3–4 PDs	−7.6	5.2	0.45	−5.2	20.4

**Table 4:** Relationships between socio-demographic variables and psychopathological symptoms in the sample of cocaine-addicted patients diagnosed with Cluster B Personality Disorder (n = 88).

	1	2	3	4	5	6	7	8	9	10	11	12	13	14	15	16
1 SCL-90-R SOM	1															
2 SCL-90-R O-C	0.77 **															
3 SCL-90-R I-S	0.56 **	0.77 **														
4 SCL-90-R DEP	0.74 **	0.89 **	0.85 **													
5 SCL-90-R ANX	0.80 **	0.79 **	0.66 **	0.77 **												
6 SCL-90-R HOS	0.56 **	0.54 **	0.47 **	0.59 **	0.63 **											
7 SCL-90-R PHOB	0.65 **	0.65 **	0.57 **	0.67 **	0.71 **	0.31 **										
8 SCL-90-R PAR	0.67 **	0.74 **	0.73 **	0.77 **	0.72 **	0.69 **	0.55 **									
9 SCL-90-R PSY	0.73 **	0.73 **	0.68 **	0.80 **	0.75 **	0.54 **	0.69 **	0.74 **								
10 SCL-90-R GSI	0.87 **	0.91 **	0.82 **	0.94 **	0.90 **	0.68 **	0.74 **	0.85 **	0.87 **							
11 No. of diagnoses	-	-	-	-	0.27 *	0.38 **	-	0.34 **	-	0.22 *						
12 Gender	-	-	-	−0.23 *	-	−0.31 **	-	-	-	-	-					
13 Age	-	-	-	-	-	-	-	-	-	-	-	0.33 **				
14 SCID-II ISTR	-	-	-	-	-	-	-	-	-	-	0.44 **	-	-			
15 SCID-II NAR	-	-	-	-	-	0.23 *	-	0.33 **	-	-	0.63 **	-	-	0.25 *		
16 SCID-II BOR	0.36 **	0.42 **	0.38 **	0.48 **	0.45 **	0.46 **	0.38 **	0.53 **	0.46 **	0.49 **	0.45 **	−0.26 *	-	-	0.22 *	
17 SCID-II ANT	-	-	-	-	-	-	-	0.27 *	-	-	0.52 **	0.22 *	-	-	0.39 **	0.22 *

Legend: * = *p* < 0.05, ** = *p* < 0.01; ANT = Antisocial Trait; ANX = Anxiety; DEP = Depression; GSI = Global Severity Index; HOS = Hostility; I-S = Interpersonal Sensitivity; ISTR = Histrionic Trait; NAR = Narcissistic Trait; BOR = Borderline Trait; O-C = Obsession–Compulsion; PAR = Paranoid Ideation; PHOB = Phobic Anxiety; PSY = Psychoticism; SCID-II = Structured Clinical Interview for DSM-IV Axis II Personality Disorders; SCL-90-R = Symptom Checklist 90-Revised; SOM = Somatization. Note: Gender was coded as 0 = female and 1 = male.

## Data Availability

The data presented in this study are available upon reasonable request from the corresponding author.

## References

[1] (2023). European Drug Report: Trends and Developments.

[2] Butler A.J., Rehm J., Fischer B. (2017). Health outcomes associated with crack-cocaine use: Systematic review and meta-analyses. Drug Alcohol Depend..

[3] Mellos E., Paparrigopoulos T. (2022). Substance use during the COVID-19 pandemic: What is really happening?. Psychiatriki.

[4] American Psychiatric Association (2022). The Diagnostic and Statistical Manual of Mental Disorders, Fifth Edition, Text Revision (DSM-5-TR).

[5] Kluwe-Schiavon B., Schote A.B., Vonmoos M., Hulka L., Preller K., Meyer J., Baumgartner M., Grünblatt E., Quednow B. (2020). Psychiatric symptoms and expression of glucocorticoid receptor gene in cocaine users: A longitudinal study. J. Psychiatr. Res..

[6] Roncero C., Comín M., Daigre C., Grau-López L., Martínez-Luna N., Eiroa-Orosa F.J., Barral C., Torrens M., Casas M. (2014). Clinical differences between cocaine-induced psychotic disorder and psychotic symptoms in cocaine-dependent patients. Psychiatry Res..

[7] Sabe M., Zhao N., Kaiser S. (2022). A systematic review and meta-analysis of the prevalence of cocaine-induced psychosis in cocaine users. Prog. Neuropsychopharmacol. Biol. Psychiatry.

[8] Moeller S.J., Fleming S.M., Gan G., Zilverstand A., Malaker P., Uquillas F.D., Schneider K.E., Preston-Campbell R.N., Parvaz M.A., Maloney T. (2016). Metacognitive impairment in active cocaine use disorder is associated with individual differences in brain structure. Eur. Neuropsychopharmacol..

[9] Hunt M.G., Rushton J., Shenberger E., Murayama S. (2018). Positive effects of diaphragmatic breathing on physiological stress reactivity in varsity athletes. J. Clin. Sport Psychol..

[10] Fernández S.D., Miranda J.J.F., Pastor F.P., Muñoz F.L. (2023). Gender and addiction and other mental disorders comorbidity: Sociodemographic, clinical, and treatment differences. Arch. Womens Ment. Health.

[11] González E., Arias F., Szerman N., Vega P., Mesias B., Basurte I. (2019). Coexistence between personality disorders and substance use disorder. Madrid study about prevalence of dual pathology. Actas Esp. Psiquiatr..

[12] González-Saiz F., Vergara-Moragues E., Verdejo-García A., Fernández-Calderón F., Lozano O.M. (2014). Impact of psychiatric comorbidity on the in-treatment outcomes of cocaine-dependent patients in therapeutic communities. Subst. Abus..

[13] Trull T.J., Sher K.J., Minks-Brown C., Durbin J., Burr R. (2000). Borderline personality disorder and substance use disorders: A review and integration. Clin. Psychol. Rev..

[14] Darke S., Duflou J., Peacock A., Farrell M., Lappin J. (2023). Characteristics and circumstances of cocaine-related completed suicide in Australia, 2000-2021. Drug Alcohol Depend..

[15] Albein-Urios N., Verdejo-Román J., Soriano-Mas C., Asensio S., Martínez-González J.M., Verdejo-García A. (2013). Cocaine users with comorbid Cluster B personality disorders show dysfunctional brain activation and connectivity in the emotional regulation networks during negative emotion maintenance and reappraisal. Eur. Neuropsychopharmacol..

[16] Albein-Urios N., Martinez-Gonzalez J.M., Lozano-Rojas O., Verdejo-Garcia A. (2014). Executive functions in cocaine-dependent patients with Cluster B and Cluster C personality disorders. Neuropsychology.

[17] Kruedelbach N., McCormick R.A., Schulz S.C., Grueneich R. (1993). Impulsivity, Coping Styles, and Triggers for Cravings in Substance Abusers with Borderline Personality Disorder. J. Pers. Disord..

[18] Roberts C.A., Lorenzetti V., Albein-Urios N., Kowalczyk M.A., Martinez-Gonzalez J.M., Verdejo-Garcia A. (2021). Do comorbid personality disorders in cocaine dependence exacerbate neuroanatomical alterations? A structural neuroimaging study. Prog. Neuropsychopharmacol. Biol. Psychiatry.

[19] Links P.S., Heslegrave R., van Reekum R. (1999). Impulsivity: Core aspect of borderline personality disorder. J. Pers. Disord..

[20] Prunas A., Sarno I., Preti E., Madeddu F., Perugini M. (2012). Psychometric properties of the Italian version of the SCL-90-R: A study on a large community sample. Eur. Psychiatry.

[21] Derogatis L.R., Svitz K.L., Maruish M.E. (1999). The SCL-90-R, Brief Symptom Inventory, and Matching Clinical Rating Scales. The Use of Psychological Testing for Treatment Planning and Outcomes Assessment.

[22] First M.B., Gibbon M., Spitzer R.L., Williams J.B.W., Benjamin L.S. (1997). Structured Clinical Interview for DSM-IV Axis II Personality Disorders (SCID-II).

[23] Spitzer R.L., Williams J.B., Gibbon M., First M.B. (1992). The Structured Clinical Interview for DSM-III-R (SCID). I: History, rationale, and description. Arch. Gen. Psychiatry.

[24] Chen K.W., Banducci A.N., Guller L., Macatee R.J., Lavelle A., Daughters S.B., Lejuez C. (2011). An examination of psychiatric comorbidities as a function of gender and substance type within an inpatient substance use treatment program. Drug Alcohol Depend..

[25] Boog M., van Hest K.M., Drescher T., Verschuur M.J., Franken I.H.A. (2018). Schema Modes and Personality Disorder Symptoms in Alcohol-Dependent and Cocaine-Dependent Patients. Eur. Addict. Res..

[26] Dulit R.A., Fyer M.R., Haas G.L., Sullivan T., Frances A.J. (1990). Substance use in borderline personality disorder. Am. J. Psychiatry.

[27] Links P.S., Heslegrave R.J., Mitton J.E., van Reekum R., Patrick J. (1995). Borderline personality disorder and substance abuse: Consequences of comorbidity. Can. J. Psychiatry.

[28] Paim Kessler F.H., Barbosa Terra M., Faller S., Stolf A.R., Peuker A.C., Benzano D., Pechansky F., Brazilian ASI Group (2012). Crack users show high rates of antisocial personality disorder, engagement in illegal activities and other psychosocial problems. Am. J. Addict..

[29] Parmar A., Kaloiya G. (2018). Comorbidity of Personality Disorder among Substance Use Disorder Patients: A Narrative Review. Indian J. Psychol. Med..

[30] Westermeyer J., Thuras P. (2005). Association of antisocial personality disorder and substance disorder morbidity in a clinical sample. Am. J. Drug Alcohol Abuse.

[31] Verheul R. (2001). Co-morbidity of personality disorders in individuals with substance use disorders. Eur. Psychiatry.

[32] Frisone F., Alibrandi A., Settineri S. (2000). Problem gambling during COVID-19. MJCP.

[33] Winkler P., Formanek T., Mlada K., Kagstrom A., Mohrova Z., Mohr P., Csemy L. (2020). Increase in prevalence of current mental disorders in the context of COVID-19: Analysis of repeated nationwide cross-sectional surveys. Epidemiol. Psychiatr. Sci..

[34] Roncero C., Daigre C., Grau-López L., Ma L.R.-C., Barral C., Pérez-Pazos J., Gonzalvo B., Corominas M., Casas M. (2013). Cocaine-induced psychosis and impulsivity in cocaine-dependent patients. J. Addict. Dis..

[35] Li Y., Simmler L.D., Van Zessen R., Flakowski J., Wan J.-X., Deng F., Li Y.-L., Nautiyal K.M., Pascoli V., Lüscher C. (2021). Synaptic mechanism underlying serotonin modulation of transition to cocaine addiction. Science.

[36] Petit A., Reynaud M., Lejoyeux M., Coscas S., Karila L. (2012). Addiction à la cocaïne: Un facteur de risque de suicide? Addiction to cocaine: A risk factor for suicide?. Presse Medicale.

[37] Rounsaville B.J. (2004). Treatment of cocaine dependence and depression. Biol. Psychiatry.

[38] Barral C., Rodríguez-Cintas L., Grau-López L., Daigre C., Ros-Cucurull E., Calvo N., Ferrer M., Roncero C. (2018). Substance-induced psychotic symptoms in Borderline Personality Disorder among substance use disorder samples in Spain. Psychiatry Res..

[39] Vorspan F., Brousse G., Bloch V., Bellais L., Romo L., Guillem E., Coeuru P., Lépine J.-P. (2012). Cocaine-induced psychotic symptoms in French cocaine addicts. Psychiatry Res..

[40] Kuzenko N., Sareen J., Beesdo-Baum K., Perkonigg A., Höfler M., Simm J., Lieb R., Wittchen H.-U. (2011). Associations between use of cocaine, amphetamines, or psychedelics and psychotic symptoms in a community sample. Acta Psychiatr. Scand..

[41] Smith M.J., Thirthalli J., Abdallah A.B., Murray R.M., Cottler L.B. (2009). Prevalence of psychotic symptoms in substance users: A comparison across substances. Compr. Psychiatry.

[42] Morton W.A. (1999). Cocaine and Psychiatric Symptoms. Prim. Care Companion J. Clin. Psychiatry.

[43] Hall K., Simpson A., O’Donnell R., Sloan E., Staiger P.K., Morton J., Ryan D., Nunn B., Best D., Lubman D.I. (2018). Emotional dysregulation as a target in the treatment of co-existing substance use and borderline personality disorders: A pilot study. Clin. Psychol..

[44] Kessler R.C., Avenevoli S., McLaughlin K.A., Green J.G., Lakoma M.D., Petukhova M., Pine D.S., Sampson N.A., Zaslavsky A.M., Merikangas K.R. (2012). Lifetime co-morbidity of DSM-IV disorders in the US National Comorbidity Survey Replication Adolescent Supplement (NCS-A). Psychol. Med..

[45] Skodol A.E., Clark L.A., Bender D.S., Krueger R.F., Morey L.C., Verheul R., Alarcon R.D., Bell C.C., Siever L.J., Oldham J.M. (2011). Proposed changes in personality and personality disorder assessment and diagnosis for DSM-5 Part I: Description and rationale. Personal. Disord..

[46] Moreno-López L., Albein-Urios N., Martinez-Gonzalez J.M., Soriano-Mas C., Verdejo-García A. (2014). Prefrontal Gray Matter and Motivation for Treatment in Cocaine-Dependent Individuals with and without Personality Disorders. Front. Psychiatry.

[47] Dick D.M., Viken R.J., Kaprio J., Pulkkinen L., Rose R.J. (2005). Understanding the covariation among childhood externalizing symptoms: Genetic and environmental influences on conduct disorder, attention deficit hyperactivity disorder, and oppositional defiant disorder symptoms. J. Abnorm. Child Psychol..

[48] Khantzian E.J. (1985). The self-medication hypothesis of addictive disorders: Focus on heroin and cocaine dependence. Am. J. Psychiatry.

[49] Khantzian E.J. (1997). The self-medication hypothesis of substance use disorders: A reconsideration and recent applications. Harv. Rev. Psychiatry.

[50] Sirigatti S., Stefanile C. (2001). Il 16PF-5 Adattamento Italiano.

[51] Nutt D., King L.A., Saulsbury W., Blakemore C. (2007). Development of a rational scale to assess the harm of drugs of potential misuse. Lancet.

[52] Ryan S.A. (2019). Cocaine Use in Adolescents and Young Adults. Pediatr. Clin. N. Am..

[53] Cacciola J.S., Alterman A.I., Rutherford M.J., McKay J.R., Mulvaney F.D. (2001). The relationship of psychiatric comorbidity to treatment outcomes in methadone maintained patients. Drug Alcohol Depend..

